# The macrophage marker translocator protein (TSPO) is down-regulated on pro-inflammatory ‘M1’ human macrophages

**DOI:** 10.1371/journal.pone.0185767

**Published:** 2017-10-02

**Authors:** Nehal Narayan, Harpreet Mandhair, Erica Smyth, Stephanie Georgina Dakin, Serafim Kiriakidis, Lisa Wells, David Owen, Afsie Sabokbar, Peter Taylor

**Affiliations:** 1 Nuffield Department of Orthopaedics, Rheumatology and Musculoskeletal Sciences, Botnar Research Centre, University of Oxford, Headington, Oxford, United Kingdom; 2 Imanova Centre for Imaging Sciences, Hammersmith, London, United Kingdom; 3 Division of Brain Sciences, Imperial College, Hammersmith, London, United Kingdom; Universite de Nantes, FRANCE

## Abstract

The translocator protein (TSPO) is a mitochondrial membrane protein, of as yet uncertain function. Its purported high expression on activated macrophages, has lent utility to TSPO targeted molecular imaging in the form of positron emission tomography (PET), as a means to detect and quantify inflammation *in vivo*. However, existing literature regarding TSPO expression on human activated macrophages is lacking, mostly deriving from brain tissue studies, including studies of brain malignancy, and inflammatory diseases such as multiple sclerosis. Here, we utilized three human sources of monocyte derived macrophages (MDM), from THP-1 monocytes, healthy peripheral blood monocytes and synovial fluid monocytes from patients with rheumatoid arthritis, to undertake a detailed investigation of TSPO expression in activated macrophages. In this work, we demonstrate a consistent down-regulation of TSPO mRNA and protein in macrophages activated to a pro-inflammatory, or ‘M1’ phenotype. Conversely, stimulation of macrophages to an M2 phenotype with IL-4, dexamethasone or TGF-β1 did not alter TSPO expression, regardless of MDM source. The reasons for this are uncertain, but our study findings add some supporting evidence for recent investigations concluding that TSPO may be involved in negative regulation of inflammatory responses in macrophages.

## Introduction

Translocator protein (TSPO) is an 18kDa outer mitochondrial membrane protein, as yet of uncertain function, although roles in steroidogenesis, apoptosis and cholesterol transport have been proposed [1]. It is increasingly being utilized as a molecular target for the *in vivo* imaging of inflammation using Positron Emission Tomography (PET), based upon its known high expression on macrophages in a variety of inflammatory diseases, including multiple sclerosis [1–3], and more recently, rheumatoid arthritis (RA) [4–5].

Macrophages are a key cellular component of the inflammatory response, having roles in phagocytosis, antigen presentation, as well as potentiation of inflammation through the production of numerous pro-inflammatory cytokines, such as tumour necrosis factor-⍺ (TNF-⍺) and interleukin-23 (IL-23) [6].

Macrophages exhibit plasticity in response to their milieu [7]. Indeed, the paradigm of two opposing ends of a spectrum of macrophage phenotypes has long been established. In this model, pro-inflammatory (or ‘classically activated’) macrophages are designated the term ‘M1’, having been activated by ‘M1’ stimuli, such as lipopolysaccharide (LPS) and interferon-ɣ (IFN- ɣ) [8]. Such macrophages produce TNF-α, and reactive oxygen species, and are associated with Th-1 cell responses, potentiating inflammation [9]. In contrast, monocytes in the presence of macrophage-colony stimulating factor (M-CSF), interleukin-4 (IL-4), glucocorticoids (such as dexamethasone) or tumour growth factor-β (TGF-β), are thought to activate to an anti-inflammatory (or ‘alternatively-activated’) M2 macrophage phenotype [8,10]. Such macrophages produce chemokines such as CCL17, and are associated with Th-2 cell responses [10].

As our knowledge of macrophage phenotype develops, assisted by transcriptional studies, there is increasing realization that ‘M1’ and ‘M2’ likely only represent two ends of a spectrum of multiple, complex macrophage phenotypes that exist *in vivo*, dependent on the combination of stimuli macrophages encounter [10,11].

There is scant literature investigating the expression of TSPO on macrophages of different phenotype. Such an investigation would not only enable more accurate interpretation of TSPO targeted *in vivo* imaging, but also potentially provide insight into any role of TSPO in macrophage biology.

The majority of investigations into TSPO expression on macrophages of different phenotype have been carried out on murine models *in vitro* [12,13]. Bonsack *et al*. undertook a histological study of microglia in a murine model of intracerebral haemorrhage. This failed to demonstrate any significant difference in TSPO expression on microglia expressing the M1 marker (CD16/32), and those expressing the M2 marker (CD206) [12]. However, immunohistochemistry may not be a truly accurate means of TSPO quantification on macrophages, since TSPO is ubiquitous [1], and further, using a single macrophage marker (e.g. CD16/32 vs CD206) may not be a precise means of classifying M1 or M2 phenotype. An additional *in vitro* study of the murine macrophage cell line, RAW 264.7 [13], demonstrated a significant increase in TSPO protein levels after 24 hours stimulation with LPS, in a dose-dependent manner as determined by an increase in the binding of the TSPO specific radio-ligand [^11^C]PBR28 [13].

However, caution is needed in translating findings of such studies to human macrophages, given the debate surrounding potential fundamental differences between human and murine macrophages [14–16]. For example, much evidence suggests the enzyme arginase 1 is well known to be expressed in murine but not human macrophages activated by the same stimuli [17–19], and there is further controversy surrounding differences in production of inducible nitric oxide synthase (iNOS) and nitric oxide (NO) on activation of human and murine macrophages by the same stimuli [14–16, 20].

Here, we undertake an investigation of TSPO expression in human monocyte derived macrophages (MDM), activated to opposing ends of the currently accepted spectrum of macrophage phenotypes [21]. We demonstrate a significant difference in TSPO expression between M1 phenotype macrophages activated by the M1 ‘pro-inflammatory’ stimuli, LPS and IFN-γ, and macrophages activated by M2 ‘reparative-state’ stimuli (IL-4, dexamethasone and TGF-β1). Human macrophage/monocytes used for the study were derived from: (i) the human monocytic cell line THP-1, originating from a paediatric patient with acute myelomonocytic leukaemia [22]; (ii) peripheral blood monocytes from healthy volunteers and (iii) RA synovial fluid derived monocytes.

## Materials and methods

### Cell culture

All cells were cultured and maintained in RPMI 1640 medium (Lonza, Biowhittaker^®,^ Belgium) supplemented with 10% endotoxin free heat-inactivated foetal bovine serum (FBS), 1% penicillin/streptomycin (Lonza, Biowhittaker^®^, Belgium). Cells were maintained in a humidified incubator at 37°C and 5% CO_2_ (New Brunswick Scientific Innova™ CO-170) unless otherwise stated.

### THP-1 monocytes

Human monocytic THP-1 cells were purchased from the American Type Culture Collection (ATCC, Maryland, USA) and were cultivated in 75cm^2^ flasks, in culture media (stated above). THP-1 cells were passaged every 4 days at a ratio of between 1:2 and 1:3, to ensure cell density was not more than 0.8–1.2x10^6^ cells/mL. Cells were used from passage numbers 4–6.

### Peripheral blood monocytes from healthy volunteers

Peripheral blood monocytes were isolated from blood cones of five healthy donors purchased from the National Blood Service (Colindale, Edgware, London, UK). Here, the peripheral blood mononuclear cell fraction was extracted using density gradient centrifugation with Histopaque (Lonza, Whittaker), and continuous counter-flow elutriation to isolate the monocyte fraction. Counter-flow elutriation is a well-established process for isolating cells of different size and has been established as a technique for isolating monocytes for decades [23–26]. It combines centrifugation (the process of sedimentation under the influence of a centrifugal force), and elutriation (the process of separation by washing), to generate fractions of cells of desired size from peripheral blood [27].

Cells isolated from the blood cone underwent centrifugal elutriation in a Beckman JE6 Elutriator (Beckman Coulter, High Wycombe, UK) to obtain monocyte-rich fractions. The centrifuge was run at room temperature and a constant speed of 2200rpm, as described in Banfalvi *et al*. [27]. In brief, cells were loaded into the elutriation chamber at an increasing flow rate, and subjected to an opposing centrifugal force compared to the flow velocity. Since the centrifugal force is constant, separation of cells according to size and granularity occurs by altering the flow of the media into the centrifuge, done via a pump. Increasing the low rate in gradual steps yields successive fractions of increasingly large or dense cells being collected. Monocytes were ejected when the flow rate was increased to 18-22mLs/minute. The percentage of monocytes in the fraction being ejected was determined by concomitant analysis of cell size and granularity using flow cytometry.

Only fractions with monocyte content over 85% (as determined by flow cytometry) were utilised in this work. Cells were counted using a Neubauer chamber haemocytometer and adjusted to a concentration of 1x10^6^ cells/mL.

### Rheumatoid Arthritis-derived synovial fluid monocytes

Aspirated synovial fluid samples from the knee joints of five patients; aged 38–65, 2 males, 3 females- with established RA (as per American College of Rheumatology updated diagnostic criteria [28]) were added into 10mL lithium heparin tubes. Full ethics approval was granted for collection of synovial fluid from NRES Committee London (reference: 07/H0706/81). Synovial fluid was diluted 1:1 with sterile phosphate buffered saline (PBS), layered over Histopaque®, and centrifuged. The intermediate cell layer was removed, labelled with CD14+ magnetic beads (Miltenyi Biotech, Germany) and sorted using Magnetic Activated Cell Sorting (MACS) as per manufacturer’s instructions (Miltenyi Biotech). The resultant monocytes were washed and resuspended in 1mL of MACS buffer. The cells were counted and adjusted to 1X10^6^ cells/mL.

### Differentiation of monocytes to macrophages

#### THP-1 MDM

To differentiate THP-1 MDM, THP-1 cells were plated at a density of 0.5x10^6^/cm^2^ (CELLSTAR^®^, Greiner Bio-One, UK), and treated with 10ng/mL of phorbol 12-myristate 13-acetate (PMA, Sigma-Aldrich, Poole, UK), for 72 hours to generate a macrophage phenotype as previously described [22].

#### Healthy peripheral blood and RA synovial fluid MDM

Monocytes from healthy peripheral blood or synovial fluid mononuclear fractions were counted and resuspended to a density of 1x10^6^ cells/mL in RPMI 1640 (Lonza, Whittaker). Macrophages were differentiated from elutriated healthy monocytes and synovial fluid-derived monocytes, by culturing cells with 100ng/mL of recombinant human M-CSF (Peprotech Inc., UK) at a concentration of 1x10^6^ cells/mL, for 7 days [29]. Thereafter, fluorescence activated cell sorting (FACS) was used to confirm monocytes had been differentiated to monocyte-derived macrophages (MDM), i.e. CD68-positive cells [30] (see S1 Fig).

On day 7, cells were treated with 10ng/mL LPS (Peprotech Inc. London) and 20ng/mL recombinant human IFN-γ (Peprotech Inc., London), for 2, 4, 6 or 24 hours, to generate M1 phenotype macrophages. To generate M2 phenotype macrophages, cells were treated with either IL-4 20ng/mL, 500nM dexamethasone, or 3ng/mL TGF-β1 for 24 hours as described in previous studies [29,31–33].

### Immunofluorescence staining

After the specified incubation period, MDM were lifted using EDTA (Sigma-Aldrich), followed by gentle scraping. Cells were washed in PBS, counted and incubated at 1 million cells/mL in 1.6% paraformaldehyde solution (Thermo-Fischer) at 4°C for 20 minutes. Using a cyto-centrifuge (Cytospin, Shandon Southern Products, Runcorn, UK), cells were spun onto slides, fixed for 15 minutes in ice cold acetone, and stored at -20°C until required. For multiple antibody immunofluorescence staining and image acquisition, protocols were adapted from Dakin *et al*. [34], using the primary antibodies rabbit anti-human TSPO at 1:1000 (LSbio), and mouse anti-human CD68 at 1:600 (Dako). Isotype control antibodies were a cocktail of mouse immunoglobulin G (IgG_1_), and rabbit immunoglobulin fraction of serum from non-immunized rabbits, solid-phase absorbed (Dako). Immunofluorescence images were acquired on a Zeiss LSM 710 confocal microscope as described previously [34].

### Real-time quantitative Polymerase Chain Reaction (qPCR)

RNA was isolated using the E.Z.N.A. EaZy RNA extraction kit, and complementary DNA (cDNA) was synthesized using 500 ng of RNA, random primers (Invitrogen), and Moloney murine leukemia virus (Promega). Quantitative PCR was performed using SYBR Green I JumpStart (Sigma-Aldrich). List and details of the primers used is provided in **Table 1**.

**Table 1:** Primers used for real-time PCR.

GENE	SENSE PRIMER	ANTI-SENSE PRIMER
***18S ribosomal RNA (18S)***	GTAACCCGTTGAACCCCA	CCATCCAATCGGTAGTAGCG
***Translocator protein(TSPO)***	GCGGCCTGGCTAACTCCTGC	AAAGCGGGAGCCCACGAAGC
***Sterol-27-hydroxylase (CYP27A1)***	ACTGCACCAGTTACAGGTGCTTTACA	CCATGTCGTTCCGTACTGGGTACT
***ATP binding cassette transporter (ABCA1)***	TTCCCGCATTATCTGGAAAGC	CAAGGTCCATTTCTTGGCTGT
***Tumour necrosis factor-alpha*** ***(TNF-α)***	GGCCAAGCCCTGGTATGAG	TAGTCGGGCCGATTGATCTC
***Prostaglandin endoperoxide synthase 2 (PTGS2)***	ATGCTGACTATGGCTACAAAAGC	TCGGGCAATCATCAGGCAC
***Interleukin 12 beta (IL-12***β***)***	AGAAGATGGTATCACCTGGACC	GAACCTCGCCTCCTTTGTGAC
***Interleukin 10 (IL-10)***	TACGGCGCTGTCATCGATT	GGCTTTGTAGATGCCTTTCTCTTG
***Transglutaminase 2*** ***(TGM2)***	GCCACTTCATTTTGCTCTTCAA	TCCTCTTCCGAGTCCAGGTACA
***Mannose receptor C type-1*** ***(MRC1)***	AAGGCGGTGACCTCACAAG	AAAGTCCAATTCCTCGATGGTG
***CD200 Receptor 1 (CD200R1)***	GACCAGAGAGGGTCTCACCA	TTGAAGCGGCCACTAAGAAG

In order to provide evidence that macrophages had been activated to an appropriate phenotype by the stimuli, mRNA expression of multiple markers were assessed by qPCR. *TNF-α*, *prostaglandin endoperoxide synthase 2 (PTGS2)*, and *interleukin-12 β (IL-12β)* mRNA are thought to be more highly expressed in M1 compared to M2 phenotype macrophages [9]. In contrast, *interleukin-10* (*IL-10)*, *Transglutaminase 2 (TGM2)*, *Mannose receptor C type-1 (MRC1)*, *and CD200 Receptor 1 (CD200R1)* mRNA have all been reported as M2 macrophage markers, demonstrated to be more highly expressed in M2 compared to M1 macrophages [35–37].

### Enzyme-Linked Immunosorbent Assay (ELISA)

It is recognized that TNF-α production in macrophages may serve as an indicator of macrophage phenotype, being higher in M1 compared to M2 phenotype macrophages [35]. Therefore, TNF-α ELISA was undertaken, to provide additional evidence of macrophage phenotype, at protein level.

The concentrations of soluble human TNF-α in cell culture media were measured using commercially-available ELISA (Quantikine human TNF-α ELISA Kits, R&D Systems Europe) according to the manufacturer’s instructions. Each reaction was run in triplicate. The optical density measurements were carried out using ELISA plate reader (Multiskan Ascent Thermo Labsystems, Finland) at 450 nm wavelength. The lower and higher detection limits of the kits were 15.6pg/mL and 1000pg/mL, respectively.

### Western blots

Total cellular protein extracts were prepared using denaturating lysis buffer [8M urea, 1% sodium dodecyl sulfate, 1% glycerol, 10 mM Tris (pH 6.8), 0.5 mM protease inhibitor cocktail (Sigma-Aldrich, containing AEBSF (4-(2-aminoethyl) benzenesulfonyl fluoride hydrochloride), aprotinin, bestatin, E-64, leupeptin and pepstatin A), 1mM dithiothreitol (Sigma-Aldrich)]. Protein extracts were separated on NuPAGE Tris-Acetate gels (4–12%; Invitrogen) and blotted onto polyvinylidene difluoride (PVDF) membranes. The membranes were incubated overnight with goat anti-human TSPO monoclonal antibody (GeneTex) at a titre of 1:10,000, or mouse anti-β-actin at 1:10,000 (Sigma-Aldrich). Following incubation with secondary polyclonal donkey anti-goat or rabbit anti-mouse antibodies (1:500 and 1:3000, respectively) coupled with horseradish peroxidase (Dako) the resultant bands were visualized using ECL Plus (GE Healthcare). Optical densitometry analysis was carried out utilizing ImageJ software (NIH), as previously described [38].

### Radio-ligand saturation binding assay

[^3^H]PBR28 is a radioligand with high selectivity and affinity for TSPO [39]. PBR28 has been used successfully in multiple imaging studies as a means of detecting TSPO protein both *in vitro* and *in vivo* [13,40,41]. To ensure all donors were high affinity binders to [^3^H]PBR28, DNA extraction and genotyping was performed for all cell donors as previously described by Owen *et al*. [42].

Due to the large volume of protein required to undertake saturation binding (minimum 2.4mg cytoplasmic protein per condition for each donor), only healthy donor peripheral blood monocytes and MDM were used for radioligand binding work. Three conditions of MDM were generated; unstimulated, and stimulated with 10ng/mL LPS plus 20ng/mL IFN-γ or with 20ng/mL IL-4, for 24 hours. Cells were pelleted, and membrane preparation, along with homogenate and competition binding assays carried out as described previously [43].

### Analysis of saturation binding data

All saturation and competition data were analysed using the iterative nonlinear regression curve-fitting software supplied with GraphPad Prism 7.0. Single-site and two-site models were compared using the least-squares algorithm. The null hypothesis, that the data fitted a single-site model, was rejected if the *p*-value was less than 0.05. Amount of tracer required (fmol/mg protein) to occupy all TSPO sites (Bmax) was calculated. Bmax value was multiplied by the total amount of protein in each cell pellet used, and then divided by number of cells in each pellet, to generate a value of fmol of [^3^H]PBR28 required to bind to all TSPO present in the cell pellet being assayed [44].

### Statistical analysis

All experiments for mRNA and Western blots were carried out in triplicate. Results are expressed as mean ± standard error of the mean (SEM). Graph Pad Prism 7.0 software was used for all data analyses. To evaluate effects of stimuli (M-CSF, LPS, IFN-γ, IL-4, TGF-β1, dexamethasone), analysis of variance (ANOVA), followed by a Bonferroni’s Multiple Comparison Test was used if there were more than two groups to compare, or Student’s *t*-test applied if two groups were analysed. *P* values less than 0.05 were considered significant (*p<0.05, **p <0.01, ***p≤0.001).

## Results

### *TSPO* mRNA is up-regulated in macrophages compared to monocytes

**Fig 1:**
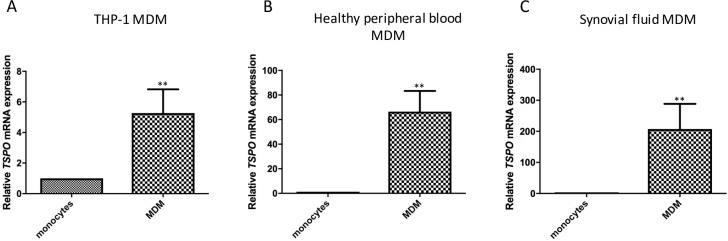
*TSPO* mRNA expression of monocytes compared to MDM. **(A)** THP-1 monocytes and THP-1 MDM; **(B)** healthy peripheral blood monocytes and corresponding MDM **(C)** synovial fluid monocytes and corresponding MDM. Results are expressed as fold change mRNA expression (compared to *18s* RNA). Each experiment was run in triplicate, with data expressed as mean ± SEM of five independent experiments. Relative *TSPO* mRNA expression in MDM was compared to their respective monocyte counterpart whereby statistically significant differences are noted as **p<0.001 using Student’s *t*-test.

*TSPO* is up-regulated on MDM compared to monocytes, even without further addition of stimuli to generate an activated macrophage phenotype (**Fig 1**). For THP-1 monocytes, addition of 10ng/mL PMA for 72 hours, resulted in an average 6-fold increase of TSPO expression (6.52 ± 1.28 fold change) (**Fig 1A**). For both healthy human peripheral blood MDM and synovial fluid MDM, monocytes were differentiated with 100ng/mL M-CSF for 7 days. Although all groups of MDM demonstrated a significant increase in *TSPO* expression compared to their monocyte counterparts (p<0.01), the magnitude of TSPO up-regulation was greater in MDM derived from the synovial fluid of arthritis patients (mean fold change of 284.6 ± 27.88 on synovial fluid MDM compared to synovial fluid monocytes, versus mean fold change 65.4 ± 16.88 for healthy peripheral blood MDM compared to healthy monocytes; **Fig 1B** and **1C**).

### TSPO protein is up-regulated on macrophages compared to monocytes

**Fig 2:**
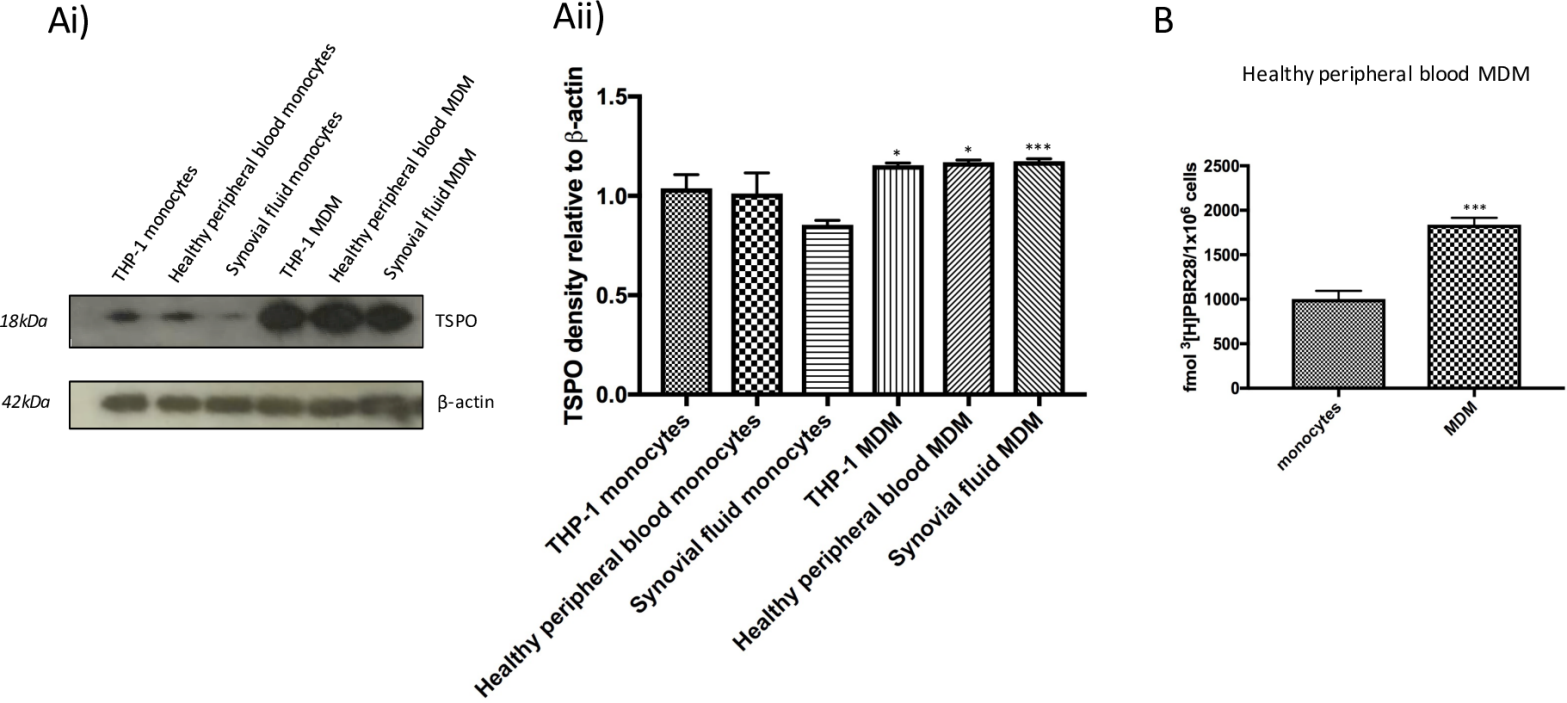
TSPO protein expression is higher in MDM than in monocytes. TSPO protein was assessed by Western blot with β-actin as loading control **(Ai)**, with accompanying densitometry normalised to β-actin **(Aii)**. Results shown are for THP-1 monocytes, healthy peripheral blood monocytes, synovial fluid monocytes, and corresponding MDM. **(B)** [^3^H]PBR28 tracer binding per 1x10^6^ cells in healthy human peripheral blood monocytes and MDM. Each experiment was run in triplicate, and data expressed as mean± SEM for five independent experiments. *** p<0.001 using Student’s *t*-test, comparing data from each MDM with that of their counterpart monocytes.

Western blot data demonstrated increased TSPO protein expression in MDM from all sources of human macrophages compared to monocytes (**Fig 2A**), with a small (p = 0.05), but significant increase in TSPO densitometry on monocyte to macrophage differentiation (**Fig 2A**). In order to provide a quantitative assessment of TSPO protein expression in monocytes compared to macrophages, [^3^H]PBR28 radioligand binding studies were undertaken. This demonstrated a significant (p<0.001) up-regulation of TSPO at protein level in healthy human peripheral blood MDM compared to monocytes (mean 1838 ± 45.37 fmol [^3^H]PBR28 per 1x10^6^ MDM, compared to mean 1004 ± 52.61 fmol [^3^H]PBR28 per 1x10^6^ monocytes) (**Fig 2B**).

### Changes in M1- & M2-specific mRNA markers in response to LPS plus IFN-γ or IL-4 stimulation

Transcriptional studies are increasingly used to accurately differentiate between macrophage phenotypes, with the expression of mRNA of *TNF-α*, *IL-12β* and *PTGS2* being higher in M1 macrophages than M2 macrophages [9,34]. In contrast, mRNA of *IL-10*, *CD200R1*, *MRC1* and *TGM2* are well-recognised to be upregulated in M2 compared to M1 macrophages [9,35–37].

**Fig 3:**
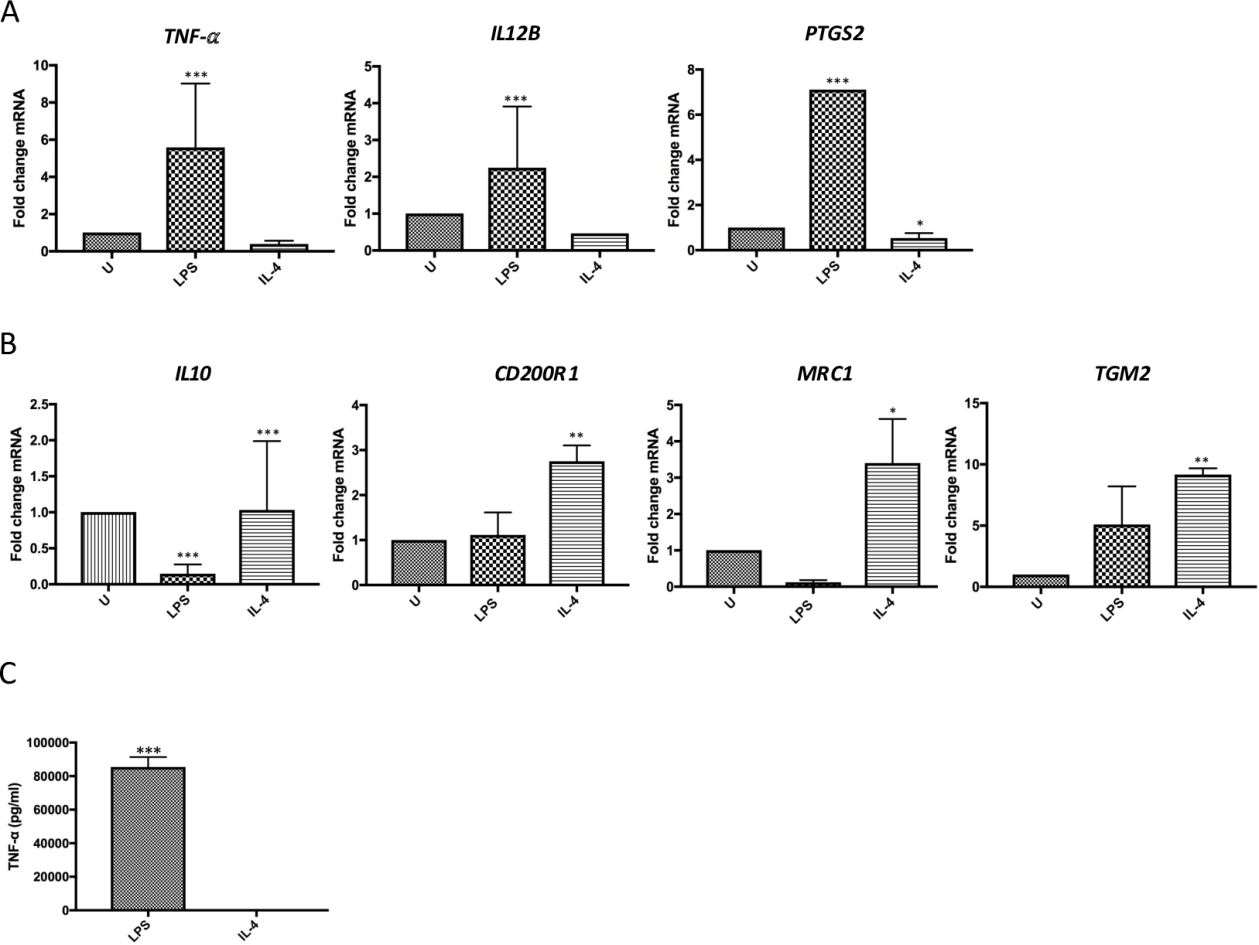
Expression of M1 & M2 markers in M1 and M2 MDM compared to untreated MDM. Expression of M1 & M2 markers relative to untreated healthy human peripheral blood MDM (U) in MDM treated with 10ng/mL LPS plus 20ng/mL IFN-γ (LPS) (M1 stimuli), or 20ng/mL IL-4 for 24 hours (IL-4) (M2 stimulus). **(A)** mRNA expression of M1 macrophage markers; *TNF-α*, *IL-12β and PTGS2*, in LPS plus IFN-γ or IL-4 treated MDM, compared to untreated MDM. **(B)** M2-specific mRNA expression of M2 macrophage markers; *IL-10*, *CD200R1*, *MRC1*, *TGM2*, in LPS plus IFN-γ- or IL-4-treated MDM, compared to untreated MDM. **(C)** TNF-α in supernatant of healthy human peripheral blood MDM stimulated with 10ng/mL LPS plus 20ng/mL IFN-γ, or 20ng/mL IL-4 for 24 hours, were measured using an ELISA Quantikine assay. Each experiment was performed in triplicate, with data expressed as mean ± SEM of five independent experiments. (*p<0.05, **p <0.01, ***p≤0.001).

Analysis of the M1 macrophage markers, *TNF-α*, *IL-12β* and *PTGS2*, revealed significantly higher expression of all these M1 markers in healthy human peripheral blood MDM treated with LPS plus IFN-γ, compared to MDM treated with IL-4 (p<0.01) (**Fig 3A**). Analysis of mRNA expression of M2 markers, *IL-10*, *CD200R1*, *MRC1* and *TGM2*, showed a significantly greater expression in healthy human peripheral blood MDM treated with IL-4 compared to MDM treated with LPS and IFN-γ (**Fig 3B**). **Table 2** demonstrates the ratio of fold change mRNA expression of each marker in MDM treated with LPS plus IFN-γ to those treated with IL-4. In addition, stimulation of human peripheral blood MDM treated with LPS and IFN-γ resulted in a significant increase in TNF-α secretion as compared to IL-4-treated cultures (85,481 ± 3452 pg/mL vs. 27.16 ± 4.17 pg/mL) (**Fig 3C**).

**Table 2:** M1:M2 ratio of mean fold change of macrophage mRNA markers.

**M1 markers**	**M1:M2 ratio**
*Tumour necrosis factor alpha (TNF-α)*	18.12
*Interleukin 12β (IL-12β)*	9.66
*prostaglandin-endoperoxide synthase 2 (PTGS2*, *also known as Cox2)*	13.40
**M2 markers**	**M1:M2 ratio**
*Interleukin-10 (IL-10)*	0.16
*CD200R*	0.41
*C-type mannose receptor 1 (MRC-1*, *also known as CD206)*	0.04
*Transglutaminase 2 (TGM-2)*	0.34

M1: M2 ratio of mean fold change of each M1 or M2 specific macrophage marker in healthy human peripheral blood MDM. M1 phenotype MDM were generated by stimulation of MDM with 10ng/mL LPS plus 20ng/mL IFNγ for 24 hours, and M2 phenotype MDM generated by stimulation of MDM with 20ng/mL IL-4 for 24 hours (*M2*). Values are expressed to nearest 2 decimal places and presented as mean fold change for 5 independent experiments.

### Immunofluorescence demonstrates no difference in staining for TSPO on MDM treated with LPS and IFN-γ and MDM treated with IL-4

**Fig 4:**
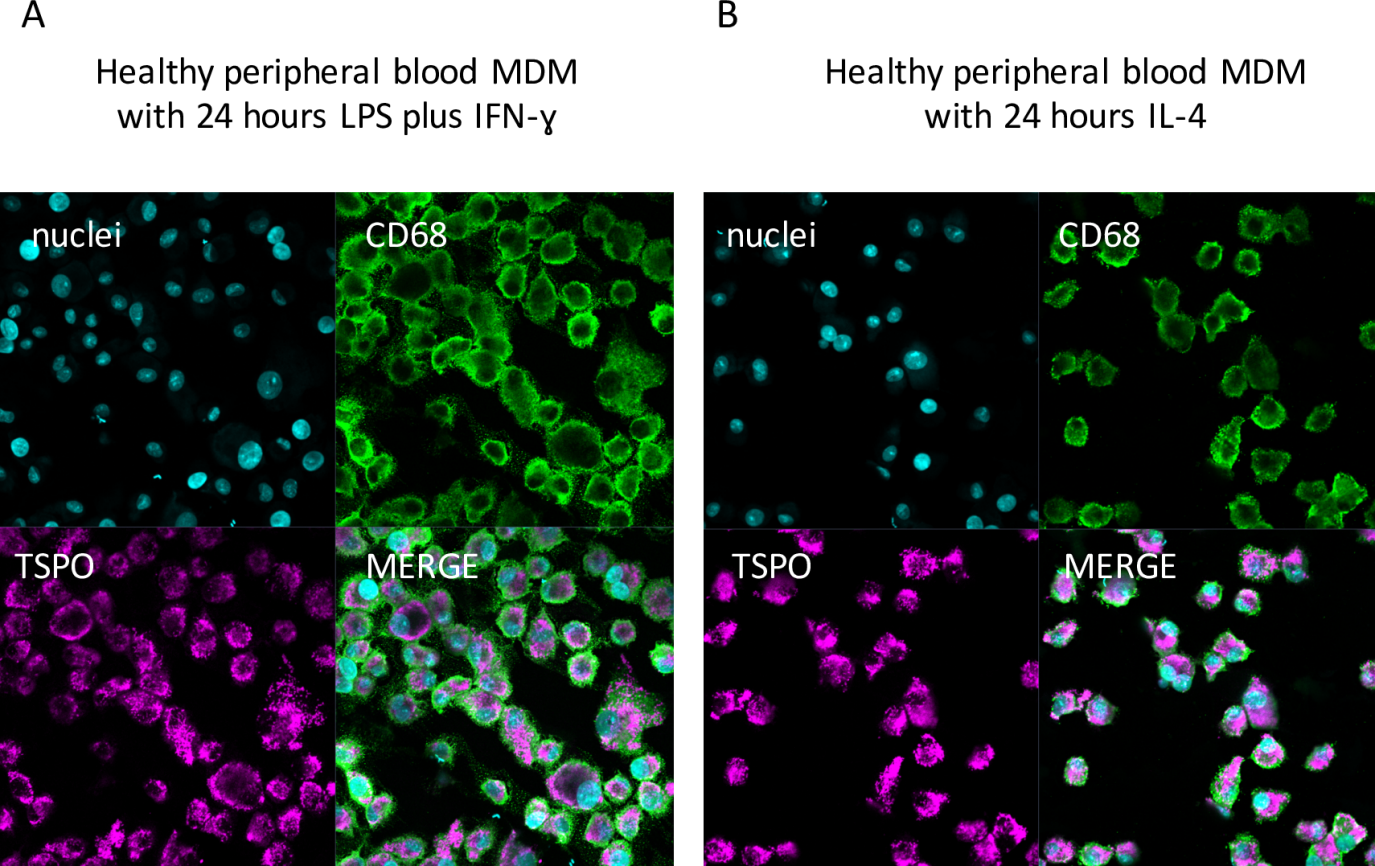
Representative immunofluorescence staining images of cytospin slides of healthy peripheral blood M1 and M2 MDM. Immunofluorescence staining of cytospin slides of healthy peripheral blood MDM with the nuclear stain POPO-1 (to confirm presence of cells), TSPO, the macrophage marker CD68 and merging of the three stains. **(A)** staining of MDM stimulated with LPS plus IFN*-*γ for 24 hours; **(B)** staining of MDM stimulated with IL-4 for 24 hours. x400 magnification. Scale bar = 20μm.

Immunofluorescence staining of MDM treated with LPS and IFN-γ (**Fig 4A**), and MDM treated with IL-4 (**Fig 4B**) indicated that all cells were CD68 positive, confirming their macrophage phenotype. Furthermore, staining for TSPO demonstrated that the CD68+ cells expressed TSPO protein irrespective if they were stimulated with LPS and IFN-γ or IL-4 (**Fig 4**).

### *TSPO* mRNA expression is reduced in M1 macrophages

**Fig 5:**
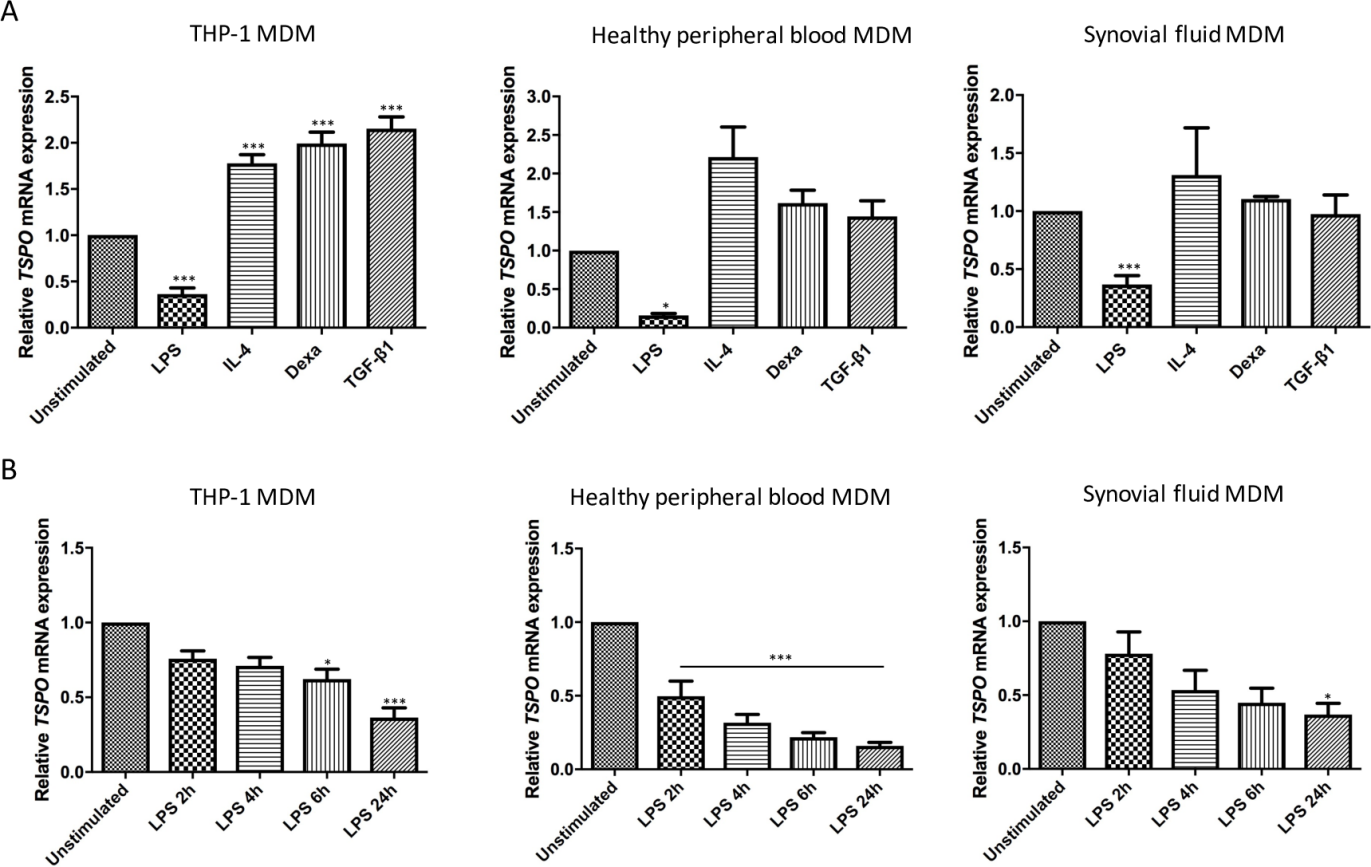
Relative *TSPO* mRNA expression in MDM from THP-1, healthy human peripheral blood, and RA synovial fluid. MDMs were stimulated with: **(A)** 10ng/mL LPS plus 20ng/mL IFN-γ, 20ng/mL IL-4, 500nM dexamethasone or 3ng/mL TGF-β1 for 24 hours, or **(B)** LPS plus IFN-γ for 2, 4, 6 and 24 hours. Each experiment was run in triplicate, with data expressed as mean of five independent experiments ± SEM and compared to unstimulated control cultures. (*p<0.05, **p <0.01, ***p≤0.001).

For MDM derived from all sources (THP-1 monocytes, healthy human peripheral blood monocytes, and synovial fluid monocytes), there was a statistically significant down-regulation of *TSPO* mRNA expression in MDM stimulated with LPS and IFN-γ, compared to both unstimulated MDM (p<0.01 in THP-1 MDM, p<0.05 in healthy human peripheral blood MDM, and p<0.01 in synovial fluid MDM; **Fig 5A**). Likewise, there was a reduction in *TSPO* mRNA expression between MDM stimulated with LPS plus IFN-γ, compared to those stimulated with IL-4, dexamethasone or TGF-β1 (p<0.01 for THP-1 MDM, healthy human MDM and synovial fluid MDM) (**Fig 5A**).

In contrast, there was no statistically significant difference between *TSPO* mRNA expression in unstimulated MDM versus MDM treated with M2 stimuli (IL-4, dexamethasone, or TGF-β1) in healthy human peripheral blood MDM or synovial fluid MDM. In THP-1 MDM, there was a statistically significant increase in *TSPO* mRNA expression in MDM stimulated with any M2 stimuli (IL-4, dexamethasone or TGF-β1) compared to unstimulated MDM (p<0.001).

In order to ensure there is no increase in *TSPO* mRNA expression in LPS plus IFN-γ stimulated MDM prior to 24 hours, a series of different stimulation durations were employed; 2,4,6 and 24 hours (**Fig 5B**). This demonstrated a reduction in *TSPO* expression over time in MDM stimulated with LPS plus IFNγ, with a statistically significant reduction in TSPO expression (relative to unstimulated MDM) being apparent as early as 2 hours for healthy human peripheral blood MDM (p<0.001), by 6 hours for THP-1 MDM (p<0.05), and by 24 hours in synovial fluid MDM (p<0.05).

### TSPO protein expression in human M1 and M2 macrophages

**Fig 6:**
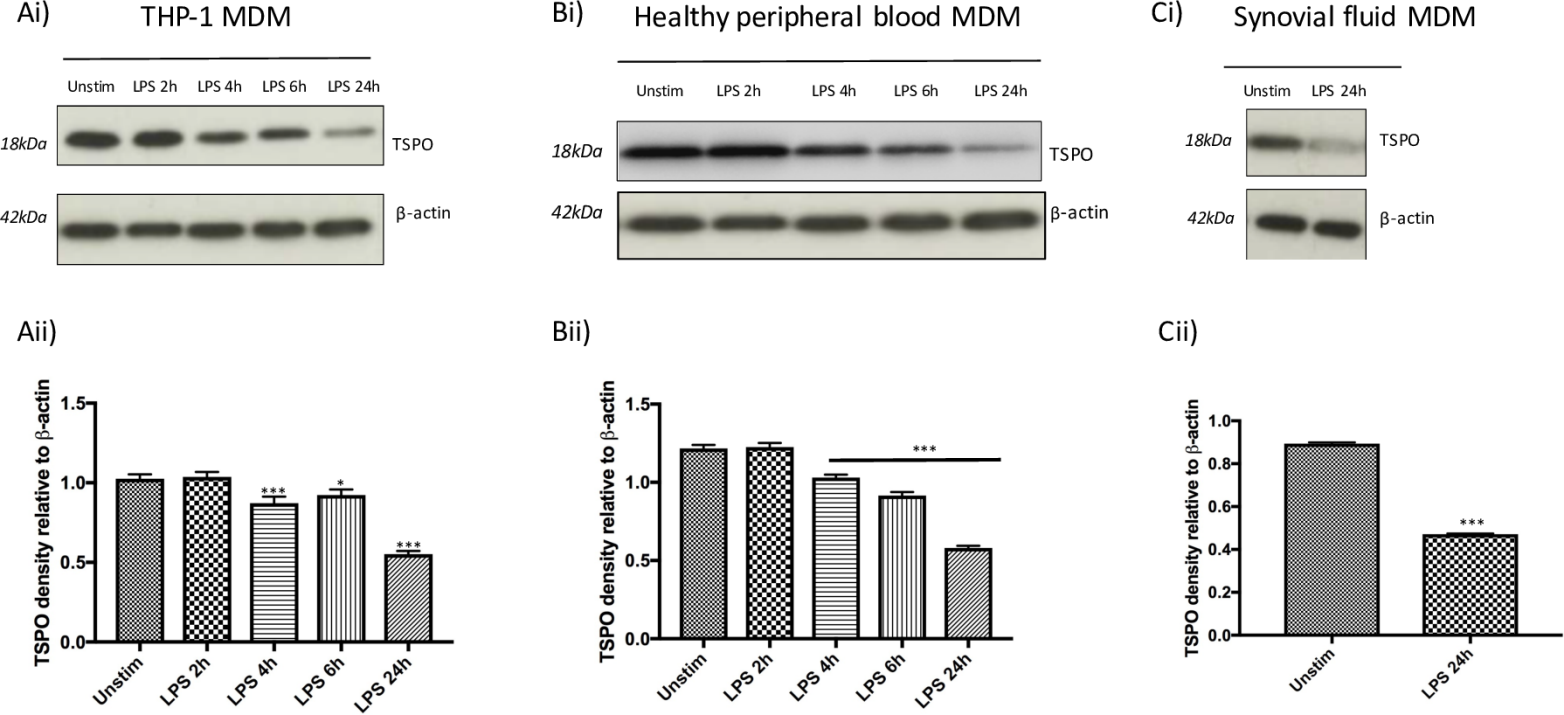
Western blotting indicates TSPO protein expression decreases on treatment of MDM with M1 stimuli LPS plus IFN-γ. (i) representative western blot of TSPO protein with β-actin acting as loading control and (ii) densitometry normalized to β-actin in (**A)** THP-1 MDM, (**B)** healthy peripheral blood MDM and (**C)** synovial fluid MDM either unstimulated (*unstim*) or treated with 10ng/mL LPS plus 20ng/mL IFN-γ (*LPS*) for 2,4,6 and 24 hours. Densitometry data is expressed as the mean of five independent experiments ± SEM. (*p<0.05, **p <0.01, ***p≤0.001).

**Fig 7:**
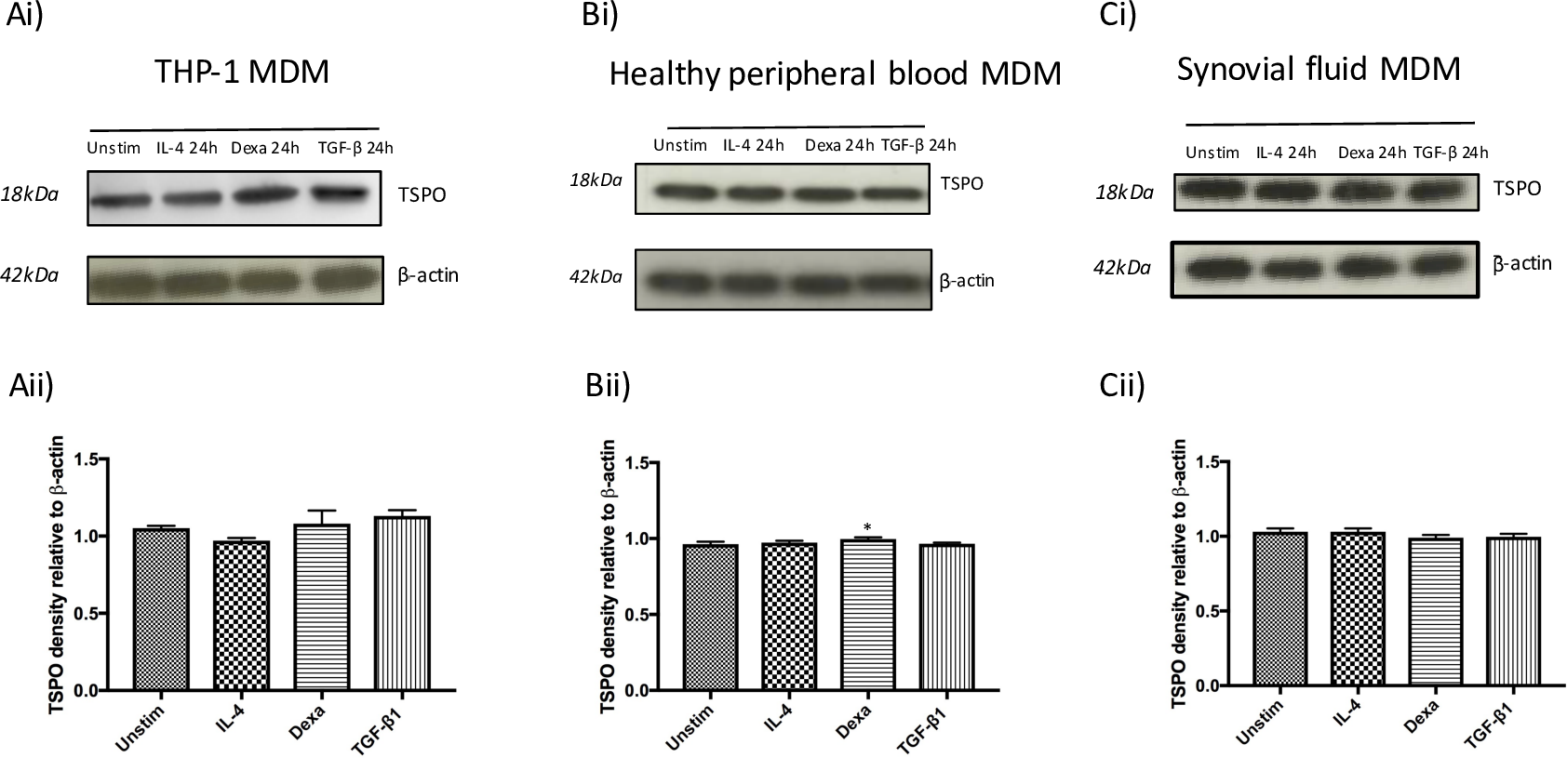
Western blotting indicates TSPO protein expression is largely unchanged in MDM treated with M2 stimuli. (i) representative Western blots of TSPO protein with β-actin acting as loading control and (ii) densitometry normalized to β-actin in **(A)** THP-1 MDM, **(B)** healthy peripheral blood MDM and **(C)** synovial fluid MDM either unstimulated (*Unstim*) treated with 20ng/mL IL-4, 500nM dexamethasone or 3ng/mL TGF-β1 for 24 hours. Densitometry data is expressed as the mean of five independent experiments ± SEM. (*p<0.05, **p <0.01, ***p≤0.001).

Results of Western blots indicated a down-regulation of TSPO protein expression in MDM stimulated with LPS plus IFN-γ at 24 hours in THP-1 MDM, healthy peripheral blood MDM, and synovial fluid MDM (**Fig 6A–6C**). Densitometry measurements of bands on western blot confirmed a significant reduction in TSPO band density on stimulation with LPS plus IFN-γ in MDM of all sources, as early as 2 hours (**Fig 6A–6C**). In contrast, protein expression of TSPO in healthy peripheral blood MDM stimulated with M2 stimuli did not appear to differ from unstimulated MDM, with only a small significant increase (p = 0.05) in TSPO band density in healthy peripheral blood MDM on stimulation with dexamethasone 500nM for 24 hours (mean ratio of 0.96 ± 0.01 in unstimulated healthy peripheral blood MDM compared to 1.00 ± 0.01 in healthy peripheral blood MDM treated with dexamethasone) (**Fig 7**).

**Fig 8:**
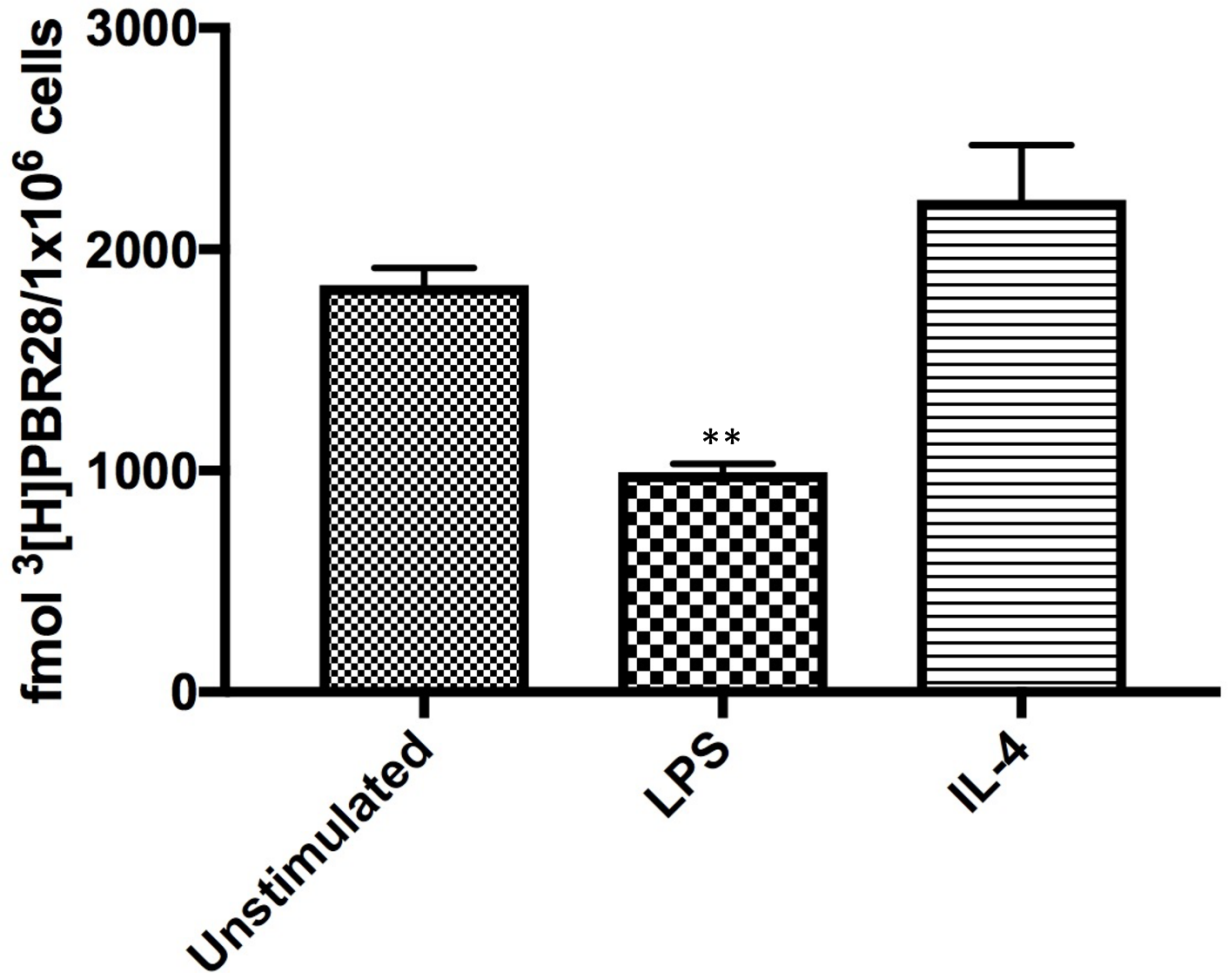
Binding of the TSPO specific radio-ligand [^3^H]PBR28 in unstimulated, M1 and M2 healthy human peripheral blood MDM. Binding of [^3^H]PBR28 in unstimulated healthy peripheral blood MDM, in MDM stimulated with LPS plus IFN-γ, and MDM stimulated with IL-4 for 24 hours. Each experiment was performed in triplicate, with data expressed as mean ± SEM of five independent experiments. ** p<0.01 compared to unstimulated.

To provide a quantitative measure of TSPO protein expression, radio-ligand binding was undertaken using the TSPO specific radio-ligand [^3^H]PBR28 (**Fig 8**). Radioligand binding demonstrated significantly less [^3^H]PBR28 binding in healthy human peripheral blood MDM stimulated with LPS plus IFN-γ compared to unstimulated MDM (p<0.01, with mean binding of 994.3 fmol [^3^H]PBR28 per 1x10^6^ MDM treated with LPS plus IFN-γ, compared to 1838 fmol [^3^H]PBR28 per 1x10^6^ unstimulated MDM). There was no significant difference between unstimulated MDM and MDM treated with IL-4 (mean binding of 2223 fmol [^3^H]PBR28 per 1x10^6^ MDM stimulated with IL-4). There was likewise a statistically significant (p<0.001) reduction of fmol [^3^H]PBR28/1x10^6^ cells between MDM stimulated with LPS plus IFN-γ compared to those stimulated with IL-4.

## Discussion

Macrophages are cells of the innate immune system and long established as key players in inflammatory diseases [45,46]. Indeed, in RA, the importance of macrophages in disease pathogenesis is well known, with infiltration of macrophages in joint tissue correlating with disease activity and joint damage [47].

The high expression of TSPO on macrophages has led to its use as a cellular target for the imaging of multiple inflammatory diseases; indeed, in the case of RA, TSPO-targeted imaging *in vivo* has been demonstrated to detect joint inflammation even before clinical signs of arthritis are apparent [48,49].

A better understanding of the expression of TSPO on macrophages of different phenotype would aid more accurate interpretation of *in vivo* TSPO targeted imaging in humans with inflammatory disease. Further, since the function of TSPO is as yet undetermined, further knowledge of TSPO expression according to macrophage phenotype, may lend insight into the potential role of TSPO in macrophage biology.

There has been scant assessment of TSPO expression on different phenotypes of human macrophages. Sandiego *et al*. attempted to ascertain the impact of the M1 stimulus, LPS, on microglia TSPO expression, by administering *Escherichia coli* LPS to healthy human volunteers, via peripheral intravenous injection [50]. TSPO PET radio-ligand [^11^C]PBR28 imaging of the brain was performed prior to, and two hours post LPS administration into peripheral blood. The authors’ data demonstrated a significant increase (nearly 2-fold on average) in [^11^C]PBR28 binding in the brain, post LPS treatment, reflecting increased TSPO expression [50]. These findings imply that LPS increases TSPO expression in the brain, but does not necessarily imply that it increases TSPO expression in microglia. Indeed, multiple cell types other than microglia exist in the brain (neurons, astrocytes and oligodendrocytes [51]) and circulating leukocytes may infiltrate brain tissue during a systemic inflammatory response [52]. Since TSPO is known to be fairly ubiquitous [53–55], it cannot therefore be assumed that the increased TSPO tracer signal on brain imaging was solely attributable to the resident microglia, and not due to contribution from other neural cells, or infiltrating cells. Further, since LPS was administered peripherally, it cannot be assumed that LPS directly up-regulated TSPO expression on microglia; indeed, imaging was undertaken at 2 hours post LPS administration, a far shorter time interval than has been previously reported for LPS to penetrate the blood brain barrier, and further LPS may not penetrate this directly [56].

Our study not only confirmed that TSPO is up-regulated on MDM compared to monocytes, but further provides the first *in vitro* data on TSPO mRNA and protein expression in human macrophages of known M1 or M2 phenotype, as defined by mRNA assessment of multiple M1 and M2 markers, as well as MDM production of TNF-α. Our data also indicate that TSPO mRNA and protein was down-regulated on MDM activated to an M1 phenotype (regardless of source), by stimulation with LPS plus IFN-γ at 24 hours. Down-regulation of *TSPO* mRNA expression was noted as early as 2 hours of treatment. The magnitude of down-regulation of TSPO protein on M1 stimulation of MDM was such that there was no significant difference between [^3^H]PBR28 binding in healthy human monocytes and healthy human M1 MDM, indicating that, the up-regulation of TSPO protein that occurs on monocyte-to-macrophage differentiation, is lost on exposure to M1 stimuli.

In contrast, there was no significant change in TSPO expression at mRNA or protein level in healthy peripheral MDM, or synovial fluid MDM activated to an M2 phenotype, compared to unstimulated MDM. Our immunofluorescence staining demonstrated TSPO expression on both M1 and M2 macrophages, in keeping with the fact that TSPO was still detectable on both macrophage phenotypes, but reinforces the point that histological staining may not be the optimal means of quantifying TSPO protein expression.

It must be noted that M1 and M2 stimuli applied in this study may not exist in isolation *in vivo*, where a combination of stimuli may act on monocytes and macrophages at any one time within a specific tissue [11,57], thus macrophage phenotypes *in vivo* may be more complex. Further study of *in vivo* human MDM harvested from different tissues, and disease states, will clarify macrophage phenotypes *in vivo*.

The reasons for a difference in TSPO expression between M1 and M2 phenotype human MDM are uncertain, given that the function of TSPO is yet to be conclusively demonstrated. However, our findings could suggest that TSPO is involved in the negative regulation of inflammation, by promoting an M2 macrophage phenotype. This hypothesis is supported by an investigation by Bae *et al*. who engineered the murine BV2 microglia cell line to overexpress TSPO [58]. This study concluded that TSPO overexpression was associated with reduced production of pro-inflammatory cytokines on LPS treatment of immortalized microglia BV2 cells, with increased expression of ‘M2’ murine macrophage markers IL-10 and MRC1, compared to microglia that did not overexpress TSPO [58].

One potential hypothesis by which TSPO expression could be associated with skewing macrophage phenotype away from the pro-inflammatory end of the spectrum, could be due to the postulated role of TSPO in cholesterol efflux [59]. A downregulation of cholesterol efflux could promote intra-cellular lipid accumulation, potentiating a pro-inflammatory (M1) macrophage phenotype, through promoting lipid raft receptor signaling, previously recognized to be an important component of pro-inflammatory signaling pathways [60,61]. Additionally, a downregulation of cholesterol efflux could promote intra-cellular cholesterol crystals accumulation, activating the inflammasome [62]. The importance of TSPO in cholesterol efflux is indicated by the fact that THP-1 derived macrophages transfected to overexpress TSPO, exhibit an increase in cholesterol efflux, whilst ligation of TSPO increased cholesterol efflux further in macrophages [63]. Silencing TSPO expression in human MDM, and investigating cholesterol efflux in *in vitro* macrophages of known M1 or M2 phenotype, would provide further confirmatory evidence to support this hypothesis.

The data presented herein have implications for the field of TSPO-targeted PET imaging, where TSPO specific radio-ligands are increasingly being used to image a range of inflammatory disease states *in vivo* [4,64–67]. In particular, in RA, TSPO-targeted PET has already been demonstrated to have potential utility for diagnosis of subclinical RA, prediction of disease flare (44, 66), and may well have utility as a tool for assessing response to therapy. With the increasing sensitivity and specificity of next generation TSPO targeted radio-ligands [5,39], it may be important to consider differences in TSPO expression in macrophages of M1 polarity compared to M2 when interpreting TSPO PET images. Indeed, given our study findings, TSPO PET itself may provide insight into the M1 or M2 polarization of macrophages in synovial tissue in RA, for which there is much conflicting data currently [46].

## Supporting information

S1 Fig Representative flow cytometry data of healthy peripheral blood monocytes and MDM. (**A**) SSC-A/FSC of harvested healthy peripheral blood monocytes. **(B)** SSC-A/FSC of unstimulated healthy peripheral blood MDM, generated after differentiation of monocytes with 100ng/mL M-CSF for 7 days. **(C)** SSC-A/PE-Cy7-A -CD68 staining of monocytes. **(D)** SSC-A/PE-Cy7-A -CD68 staining of unstimulated healthy human MDM. SSC-A = side scatter area (measure of cell granularity), FSC-A = forward scatter area (measure of cell size).
S1A and B Figs demonstrate clear increase in granularity of cells, consistent with monocyte differentiation to macrophages. S1C and D Figs demonstrate a clear increase in CD68 expression in macrophages (D) compared to monocytes (C), along with an increase in cell granularity, consistent with successful monocyte to macrophage differentiation.
(TIF)
