# Supplementary figures and images for: The macrophage marker translocator protein (TSPO) is down-regulated on pro-inflammatory ‘M1’ human macrophages
Source: PLoS One. 2017 Oct 2;12(10):e0185767. doi: 10.1371/journal.pone.0185767 (PMC5624624; doi:10.1371/journal.pone.0185767)

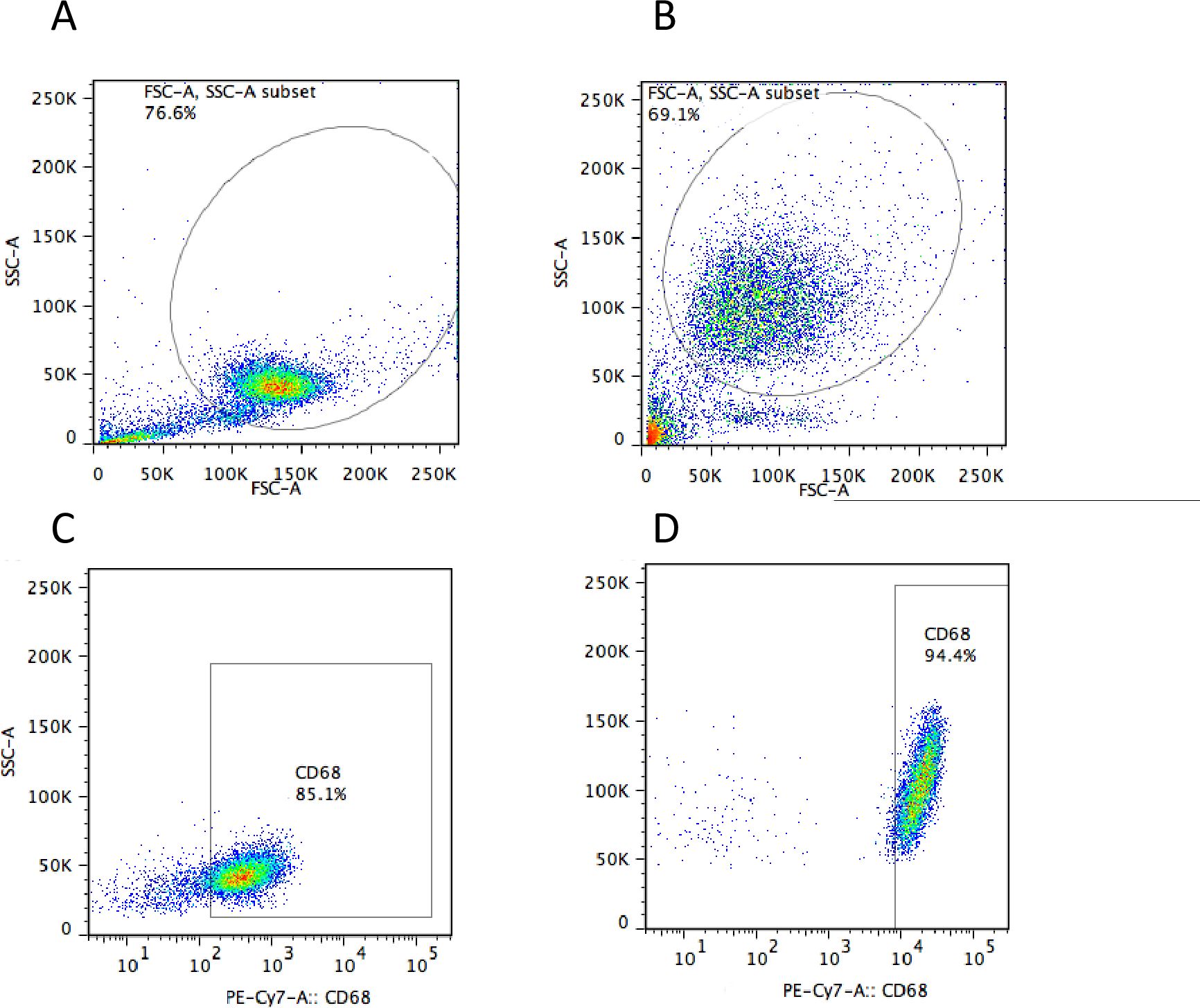

Supplement: S1 Fig — (A) SSC-A/FSC of harvested healthy peripheral blood monocytes. (B) SSC-A/FSC of unstimulated healthy peripheral blood MDM, generated after differentiation of monocytes with 100ng/mL M-CSF for 7 days. (C) SSC-A/PE-Cy7-A -CD68 staining of monocytes. (D) SSC-A/PE-Cy7-A -CD68 staining of unstimulated healthy human MDM. SSC-A = side scatter area (measure of cell granularity), FSC-A = forward scatter area (measure of cell size). S1A and B Figs demonstrate clear increase in granularity of cells, consistent with monocyte differentiation to macrophages. S1C and D Figs demonstrate a clear increase in CD68 expression in macrophages (D) compared to monocytes (C), along with an increase in cell granularity, consistent with successful monocyte to macrophage differentiation. (TIF) [file pone.0185767.s001.tif]
